# Comparing avian species richness estimates from structured and semi-structured citizen science data

**DOI:** 10.1038/s41598-023-28064-7

**Published:** 2023-01-21

**Authors:** Fang-Yu Shen, Tzung-Su Ding, Jo-Szu Tsai

**Affiliations:** 1 School of Forestry and Resource Conservation, National Taiwan University, Taipei City, Taiwan; 2 Oak Creek Lab of Biology, Department of Fisheries, Wildlife and Conservation Sciences, Oregon State University, Corvallis, OR USA; 3 College of Bio-Resources and Agriculture, The Experimental Forest, National Taiwan University, Nantou County, Taiwan; 4 Department of Biological Resources, National Chiayi University, Chiayi City, Taiwan

**Keywords:** Biodiversity, Community ecology

## Abstract

Citizen science, including structured and semi-structured forms, has become a powerful tool to collect biodiversity data. However, semi-structured citizen science data have been criticized for higher variability in quality, including less information to adjust for imperfect detection and uneven duration that bias the estimates of species richness. Species richness estimators may quantify bias in estimates. Here, we test the effectiveness of Chao1 estimator in eBird (semi-structured) by comparing it to averaged species richness in Breeding Bird Survey Taiwan, BBS (structured) and quantifying bias. We then fit a power function to compare bias while controlling for differences in count duration. The Chao1 estimator increased the species richness estimates of eBird data from 56 to 69% of the average observed BBS and from 47 to 59% of the average estimated BBS. Effects of incomplete short duration samples and variability in detectability skills of observers can lead to biased estimates. Using the Chao1 estimator improved estimates of species richness from semi-structured and structured data, but the strong effect of singleton species on bias, especially in short duration counts, should be evaluated in advance to reduce the uncertainty of estimation processes.

## Introduction

Biodiversity loss impacts ecosystem services and ecosystem functions worldwide^1^. Most recently, the loss of biodiversity has been driven by climate change, habitat conversion and fragmentation, and introduction of invasive species^2–5^. Under these impacts, it has become crucial for scientists to develop methods to monitor biodiversity across different temporal and spatial scales. Species richness, one of the most common measures of biodiversity, is defined as the number of species in a given area^6,7^. Unfortunately, monitoring species richness is well known for being expensive and labor-intensive, and often beyond the means of modestly funded research studies. In contrast, citizen science has recently emerged as an alternative that provides a low-cost approach to collect species richness data.

Citizen science projects invite volunteers to participate in and contribute observations for scientific purposes^8^. One of the biggest advantages of citizen science projects is that it generates a large number of observations, which often involve documenting species richness and species composition. Citizen science can be grouped into three main categories: structured, semi-structured, and unstructured citizen science^9^. Structured citizen science adheres to a rigorous data collection methodology and aims to produce higher quality data, by standardizing the quality of observations from volunteer training, survey duration, and choice of sampling locations^6^. Despite the higher quality output from structured citizen science programs, such as Breeding Bird Survey Taiwan (BBS), acquiring such data is not timely in most cases, because some observations need to be organized and validated before release to public. On the other hand, semi-structured citizen science usually provides options for observers to collect information^9^ and users can immediately access information. Semi-structured citizen science projects such as eBird usually allow observers to survey without time or location restrictions and data can be contributed by observers of all skill levels^6^. Yet, the abundant observations distributed across large spatial and temporal scales may be a strength of semi-structured citizen-science data. Unstructured citizen science has the least restriction regarding data collection methodology, such as iNaturalist^9^. However, unstructured citizen science often lacks important observation processes that are critical for scientific studies. Thus, in this study, we exclude unstructured citizen science due to the limitation of information to help us address the goal of this study.

Even with various efforts in standardization and training, both structured and semi-structured citizen science data may still suffer from imperfect species detectability^10–12^. Species detectability is defined as the probability to record at least one individual of a species in a given area during a fixed period of time assuming a species is present^13^. Unfortunately, species detectability is unlikely perfect, where species detectability is usually less than one; hence, due to imperfect detectability, a complete survey of species, especially animals, in an area is almost impossible to achieve^14^. Variable identification skills among volunteers that link to species detectability may introduce biased observations, resulting in recurring bias throughout the detection process, and is common in structure and semi-structured citizen science^11,12,15^. Despite rigorous survey methods and recruiting more experienced volunteers from structured citizen science, it also suffers from imperfect species detectability that may overlook undetected species^16^. Yet, few studies, especially in semi-structured and structured citizen science, have attempted to account for imperfect species detectability; thus, the biased estimate of species richness often leads to underestimation of true species richness^17^. It has been found that using observed species richness solely as an index, leads to the worst performance when compared to other species richness estimation methods^18^.

Species richness estimators attempt to estimate true species richness in a community. For example, the Chao1 estimator, a non-parametric estimation approach makes no assumptions about species abundance distribution or species detectability (i.e., heterogeneity detectability among species)^17^. Chao1 estimates the lower bound of the species richness asymptote based on the calculation of the probability of looking for a new species that is approximately equal to the proportion of rare species present in an assemblage^19^. Thus, Chao1 asserts undetected species as a means of the numbers of singletons and doubletons (i.e., rare species) to estimate the lower bound of species richness. Since the Chao1 estimator largely depends on sampling effort and sample completeness, if a sample is under-surveyed that may due to imperfect detectability from different volunteers or has a limited duration of survey, the Chao1 estimator is more likely to find undetected species and thus better estimate the lower bound of the species richness^17^. Among non-parametric approaches, the Chao1 estimator was found to be the least biased and has been widely applied in ecological studies^20,21^.

Uneven duration of effort is the most common signature of semi-structured citizen-science^8^, which strongly influences the number of recorded species^7,15,17^; thus, uneven duration of surveys may induce incomplete samples. Although technologies such as rarefaction have been applied to accommodate the bias in richness estimates, few studies have tried to compare species richness measures from different communities under a standardized duration^21^. Walther, et al.^22^ found that using uneven duration in each host species could cause a pseudo-positive correlation between duration and parasite species richness when they tried to compare species richness from different communities. Once reference samples are standardized by using even duration, a comparison among different communities on species richness measures would be more informative and accurate^23^. While semi-structured citizen science provides abundant observations with a variety of duration, steps should be taken in understanding the relationship between duration and species richness in advance before comparing species richness from even duration. In general, as sampling effort increases, observed species richness tends to approach true species richness^24^. It has been found that using a linear relationship to illustrate the effect of duration on species richness could be misleading^22^. A more robust approach such as non-linear function could be used to investigate the relationship between sampling effort and species richness (i.e., power function)^25–27^. In addition, the property of power function indicates that as the sampling effort increases, the species richness will gain monotonically. Regarding semi-structured citizen science studies, the pattern of species richness along the sampling effort adopted from variable duration is still little explored.

Although some studies have attempted to test the reliability of the accuracy of semi-structured citizen science data by comparing them to a more professional monitoring citizen science program^6,18,20,21^, few studies have taken variable duration into account when comparing datasets. Bias, an accuracy measurement, calculates the closeness from reference data to an accepted value, or true species richness^18,21^. Compared with semi-structured citizen science data, professional data tends to have a more complete inventory of focal species richness due to replicate surveys from multiple points or visits^28^. Thus, professional data can be regarded as a silver standard when comparing datasets. We adhere to regard BBS as a silver standard rather than a golden standard because BBS surveys may still be incomplete that can unlikely represent true species richness. Finally, once the effect duration has on bias has been measured by using a power function, one could compare the bias of species richness produced from semi-structured citizen science to that of structured citizen science data on a standardized time of duration.

The objective of this study was to assess the effectiveness of a species richness estimator in semi-structured citizen science data (eBird) by comparing it to structured citizen science data (BBS). We aggregated species richness from BBS dataset over eight years that followed rigorous survey methods, therefore can be considered as structured citizen science data. After applying the Chao1 estimator in both eBird and BBS datasets, we then calculated the bias measurement from the two datasets and examined the bias at a standardized duration from a power function model.

## Methods

### Citizen science datasets

Our study focused on two citizen science datasets: the Breeding Bird Survey Taiwan (BBS) and the 2017 eBird Reference Dataset (https://ebird.org/data/download) in Taiwan (from 119° 59ʹ 48.82ʹʹ E to 122° 0ʹ 26.97ʹʹ E; from 21° 53ʹ 44.16ʹʹ N to 25° 18ʹ 10.10ʹʹ N), an area of approximately 36,000 km^2^ with the highest elevation of 3,952 m a.s.l. To capture the breeding season of birds, observations from both datasets were only extracted from March to July yearly from 2010 to 2017. We only selected bird species that regularly breed in Taiwan during the breeding season. The migratory statuses of bird species followed the 2020 Checklists of Birds of Taiwan, Taiwan Wild Bird Federation^29^.

**Figure 1:**
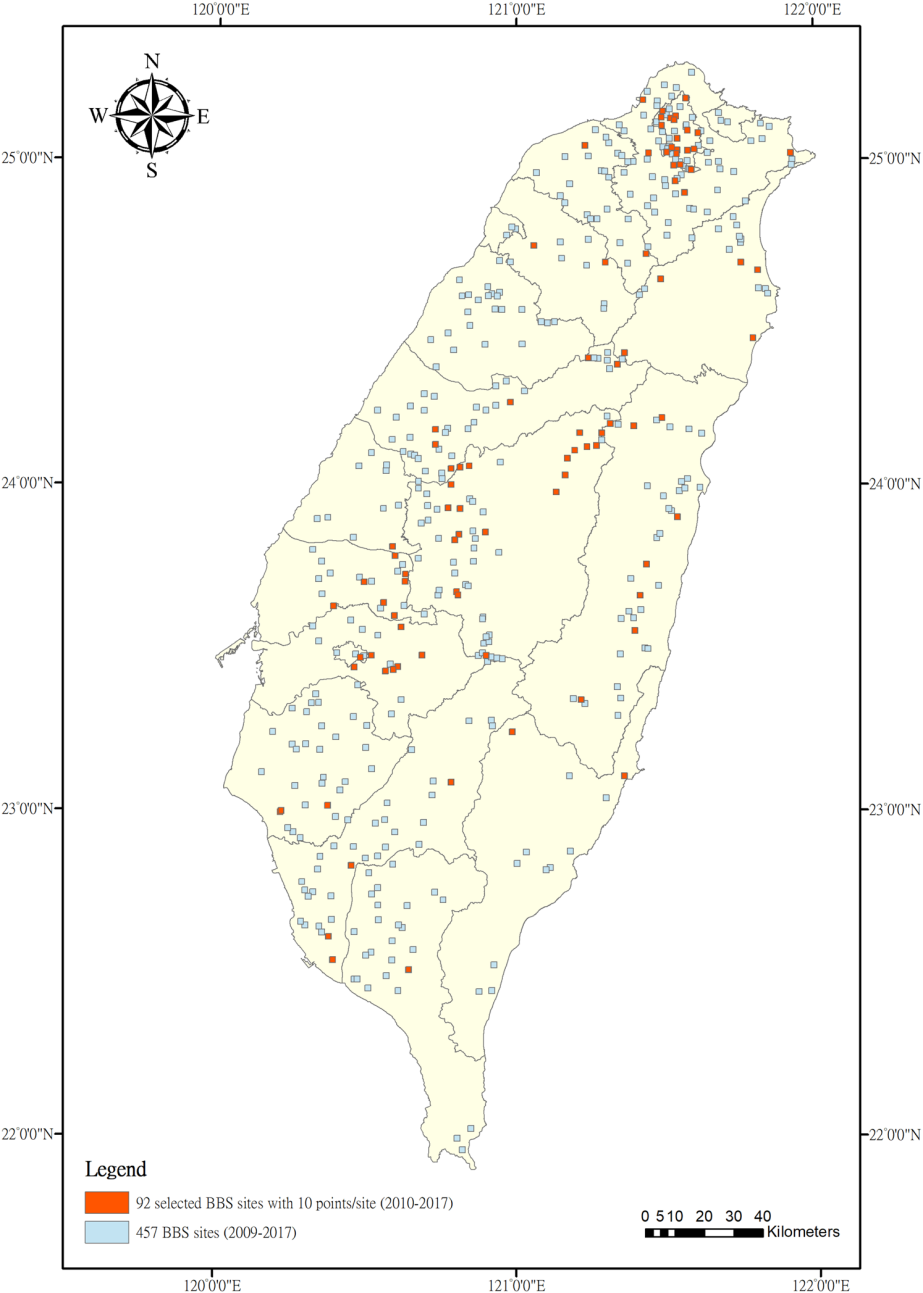
Distribution of selected 92 BBS sites (orange-colored) out of the original 457 BBS sites across Taiwan island. Solid black lines indicate the border of each county in Taiwan. Figure is drawn using ArcGIS 10.6. (http://www.esri.com/software/arcgis).

The Breeding Bird Survey Taiwan (https://sites.google.com/a/birds-tesri.twbbs.org/bbs-taiwan/bbs-zi-liao-shen-qing), led by Endemic Species Research Institute in Taiwan, has been conducted since 2009. The BBS monitoring program relies on trained volunteers to collect observations. A total of 457 sites were located across Taiwan. Each site was visited twice a year from March to July, depending on the elevation to match the peak breeding season. The surveys of each site were conducted by point counts from local sunrise to 4 hours after local sunrise on good weather conditions. Each site included 6 to 10 points located within an area of 2 × 2 km, and each point was spaced at least 200 m apart. The volunteer counted and recorded the number of all the birds heard or seen for six minutes at each point in three distance bands (0–25, 25–100, and > 100 m). We selected bird records from 2010 to 2017, since 2009 data followed a different survey protocol. Most breeding birds use fewer coastal habitats for breeding. Thus, we removed BBS sites that were intersected with the coastline. We filtered sites that included 10 points to standardize the total duration of each visit at 60 min (6 min in each of the 10 points) and excluded > 100 m of observations from each point. A total of 92 sites remained after the filtering (Fig. 1). Among the 92 BBS sites, 55 (60%) sites were defined as forest habitat, 20 (22%) as agriculture habitat, 11 (12%) as human developed habitat, 3 (3%) as grassland habitat, and 3 (3%) as wetland habitat (Supplementary Table S2 online). The BBS dataset contained a total of 1,010 visits, and 135 diurnal resident and summer visitor bird species across 92 selected sites (see Supplementary Table S1 online). Additional information regarding the sampling effort of the selected sites is shown in Supplementary Table S2 online.

eBird is a large citizen science project managed by Cornell Lab of Ornithology. Bird observations are recorded by observers following semi-structured procedures. From the 2017 eBird Reference Dataset, we selected the completed and approved checklists that were intersected within a 2 × 2 km square buffer based on centroid point from the selected 92 BBS sites with ArcGIS 10.6^10^. Hence, eBird checklists that did not fall in the buffer were excluded from the eBird dataset. Some BBS sites were close to each other geographically, creating overlapped buffers. Therefore, it is possible to have an eBird sampling location overlap with several BBS square buffers. If any eBird sampling location was intersected from two or more BBS 2 × 2 km square buffers, we treated eBird checklists separately belonging to each BBS site. Because volunteers conducted BBS may submit their BBS records to eBird, we excluded duplicated eBird checklists with location names that had similar patterns to BBS sites, such as “BBS-A35-19.” We also removed eBird checklists that contain the same coordinates (i.e., latitude and longitude) as BBS points.

We selected checklists from the three most common survey protocols in the eBird dataset: stationary, traveling, and historical. Stationary survey protocol requires a observer to remain at a fixed location; traveling survey protocol involves observers moving 30 m or more away from their starting location; historical survey protocol may consist of historical bird watching records, such as Taiwan Bird Record of Taiwan Wild Bird Federation^30^. To make eBird checklists comparable with BBS dataset, we aligned eBird checklists with the following criteria: (1) we filtered checklists that were submitted from local sunrise to 4 hours after local sunrise (local sunrise was performed by the “sunrise” function in “bioRad” package^31^); (2) we restricted checklists submitted between March and July from 2010 to 2017 to match the same timeframe as BBS; (3) we restricted a total <  = 4 km distance traveled^32^. Although few studies^10,33^ indicate that checklists with a traveling distance less than 8 km are suitable for bird studies, it may not reflect the habitat within 2 × 2 km square buffers from BBS sites in Taiwan. Thus, we used 4 km as a cut-off distance to best match habitat buffers from BBS sites; (4) we removed checklists with less than 6 min of duration because we regard a complete checklist should conduct at least 6 min of survey same as BBS point. To minimize misleading results of species richness estimation in subsequent analyses, we did the following data culling: (1) we removed the whole checklist if any bird species was reported as “X” (no specific individual count) throughout the study^33^; (2) we removed species independently from a checklist that contained missing observation (e.g. “NA”) instead of a complete count^34,35^; (3) we removed duplicated checklists, which were usually shared by individuals of same birding group, based on the sampling event identifier^36^. A total of 144 diurnal resident and summer visitor bird species and 564 checklists that fell within BBS sites were collected for further analyses (see Supplementary Table S1 online). The median number of eBird checklists per BBS site is 14.

### Statistical analysis

#### Comparing eBird and BBS datasets with and without species richness estimation

eBird checklists have variable duration and derive from many observers, both of which can generate biased richness estimates. To address bias that stems from the survey process, based on the selected 564 eBird checklists that fell within the BBS sites, we applied an abundance-based estimator, Chao1^17,23,37^ in each eBird checklist to obtain comparable measures of species richness. Although BBS comprised 10 points of surveys from 2010 to 2017, it is still possible volunteers may overlook bird species. Thus, we also applied Chao1 in each BBS survey to better capture the true species richness from a site.

To evaluate the effectiveness of applying Chao1, we compared before and after the Chao1 estimator was implemented in eBird to the average observed and estimated species richness from BBS. The average species richness from BBS was implemented because: (1) We were interested in comparing eBird and BBS with equal count durations (i.e., 60 min); a 60-min period includes 10 6-min point counts from each BBS site. (2) The compiled species richness collected through 10 points yearly in each BBS site from 2010 to 2017, regardless of the implementation of Chao1, we assume they could likely represent a standard against which eBird species richness estimates were compared. Chao1 estimation was performed using the “iNEXT” package^38^ for each eBird checklist and each BBS visit separately in the R platform ver. 4.2.0^39^. The following sections introduce the discrepancy measurement between eBird and BBS, including before and after the implementation of Chao1 in eBird and BBS:

#### Relative variance of species richness in eBird and BBS datasets

To calculate the discrepancy of species richness between the two datasets, we calculated the bias of the measurement from the observed species richness from each eBird checklist vs. the average observed species richness from each visit to the assigned BBS site (i.e., the average number of species recorded in each visit of BBS). Bias was calculated by the following formula^18,21^:

1 $${\text{Bias}} \, = \frac{{\left[ {{{O}}_{{{{ij}}}} - {{A}}_{{{i}}} } \right]}}{{\left[ {{{A}}_{{{i}}} } \right]}}$$

where *i* = 1 to 92 (refers to the *i*th BBS site); where *j* = eBird checklists in the *i*th BBS site (i.e., *j*th sample in each BBS site). *O*_*ij*_ is the observed species richness in each eBird checklist; *A*_*i*_ is the average observed species richness from each visit in the *i*th BBS site recorded from 2010 to 2017.

We then calculated the bias value from estimated species richness from each eBird checklist vs. the average observed species richness from each visit to the belonging BBS site. The outcome of bias after the Chao1 estimation was calculated using the following formula^18,21^:

2 $${\text{Bias}} = \frac{{\left[ {E_{{ij}} - A_{i} } \right]}}{{\left[ {A_{i} } \right]}}$$

where *i* = 1 to 92 (refers to the *i*th BBS site); where *j* = eBird checklists in the *i*th BBS site (i.e., *j*th sample in each BBS site). *E*_*ij*_ is the outcome of estimated species richness derived from the Chao1 in each eBird checklist; *A*_*i*_ is the average observed species richness from each visit in the *i*th BBS site recorded from 2010 to 2017.

To better capture the true species richness in BBS sites, we implemented the Chao1 in each BBS site and calculated the bias of the measurement from the observed species richness from each eBird checklist vs. the average estimated species richness from each visit to the assigned BBS site (i.e., the average number of species estimated in each visit of BBS). Bias was calculated by the following formula^18,21^:

3 $${\text{Bias}}{\kern 1pt} = \frac{{\left[ {O_{{ij}} - B_{i} } \right]}}{{\left[ {B_{i} } \right]}}$$

where *i* = 1 to 92 (refers to the *i*th BBS site); where *j* = eBird checklists in the *i*th BBS site (i.e., *j*th sample in each BBS site). *O*_*ij*_ is the observed species richness in each eBird checklist; *B*_*i*_ is the average estimated species richness from each visit in the *i*th BBS site recorded from 2010 to 2017.

Finally, we calculated the bias value from estimated species richness from each eBird checklist vs. the average estimated species richness from each visit to the belonging BBS site. The following formula represents the outcome of bias after the Chao1 was implemented in eBird checklists and BBS visits^18,21^:

4 $${\text{Bias}}{\kern 1pt} = \frac{{\left[ {E_{{ij}} - B_{i} } \right]}}{{\left[ {B_{i} } \right]}}$$

where *i* = 1 to 92 (refers to the *i*th BBS site); where *j* = eBird checklists in the *i*th BBS site (i.e., *j*th sample in each BBS site). *E*_*ij*_ is the outcome of estimated species richness derived from the Chao1 in each eBird checklist; *B*_*i*_ is the average estimated species richness from each visit in the *i*th BBS site recorded from 2010 to 2017.

#### Evaluating the effectiveness of species richness estimator

To assess the effect of duration on survey bias from the eBird dataset vs. BBS dataset at the duration of 60 min, we fitted a power function^25^ by using the least squares method^40^ across all included 564 eBird checklists. The power function is used to estimate the monotonic increment properties of species richness as duration increases^40,41^. We treated duration in each eBird checklist as an independent variable; bias derived from observed and estimated species richness was treated as a dependent variable separately. We adjusted the power function by adding “$- 1$” to better interpret the completeness of eBird compared to BBS. Finally, we calculated a 95% confidence interval with 10,000 simulations for the fitted value from the power function by using first-/second-order Taylor expansion and Monte Carlo simulation in the “propagate” package^42^. The formula of power function where bias is derived from observed species richness of eBird vs. average observed species richness of BBS is depicted as follows:

5 $$y_{1} = ax^{b} - 1$$

where, *y*_*1*_ is the bias derived from Eq. (1), as the dependent variable, and *x* is the duration, as the independent variable; *a*, *b* denote the parameters to be estimated by the least squares method^40^.

The formula of power function where bias is derived from estimated species richness of eBird vs. average observed species richness of BBS is depicted as follows:

6 $$y_{2} = cx^{d} - 1$$

where, *y*_*2*_ is the bias derived from Eq. (2), as the dependent variable, and *x* is the duration, as the independent variable; *c*, *d* denote the parameters to be estimated by the least squares method^40^.

The formula of power function where bias is derived from observed species richness of eBird vs. average estimated species richness of BBS is depicted as follows:

7 $$y_{3} = ex^{f} - 1$$

where, *y*_*3*_ is the bias derived from Eq. (3), as the dependent variable, and *x* is the duration, as the independent variable; *e*, *f* denote the parameters to be estimated by the least squares method^40^.

The formula of power function where bias is derived from estimated species richness of eBird vs. average estimated species richness of BBS is depicted as follows:

8 $$y_{4} = gx^{h} - 1$$

where, *y*_*4*_ is the bias derived from Eq. (4), as the dependent variable, and *x* is the duration, as the independent variable; *g*, *h* denote the parameters to be estimated by the least squares method^40^. The parameters estimation above were calculated with the “stats” package^43^ in the R platform.

Our goal was to compare the resulting estimation from ${\varvec{y}}_{1}$ to ${\varvec{y}}_{2}$**,** as well as from ${\varvec{y}}_{3}$ to ${\varvec{y}}_{4}$ at duration for which the source provides enough information to test the effectiveness of the Chao1 estimation. We then set a 60-min to standardize the comparison of bias before and after the implementation of the Chao1 estimator. Finally, we summarize the discrepancy between ${\varvec{y}}_{1}$ vs. ${\varvec{y}}_{2}$, and ${\varvec{y}}_{3}$ vs. ${\varvec{y}}_{4}$ through their relative proportion of species richness derived from Eqs. (1–4).

## Results

**Figure 2:**
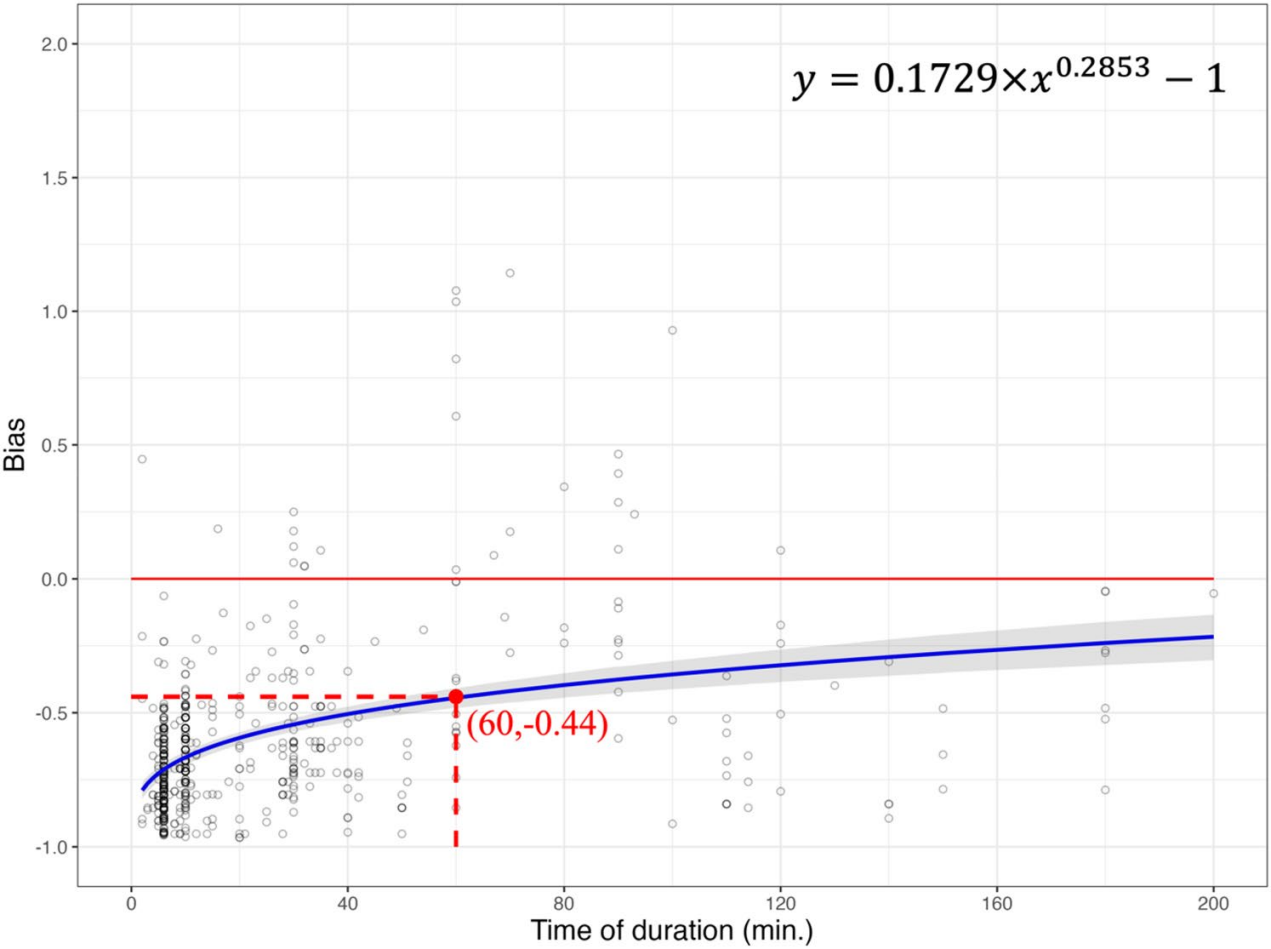
The relationship of duration on eBird checklists and bias. The power function (formula on the top right) estimated the non-linear relationship between duration and bias (blue solid line). Bias was derived from the results of observed species richness in eBird and average observed species richness from BBS (black circle). Where bias = 0 (red solid horizontal line) indicates no differences among observed species richness from eBird and average observed species richness from BBS, indicating eBird could record an average of 100% of the BBS species richness.

**Figure 3:**
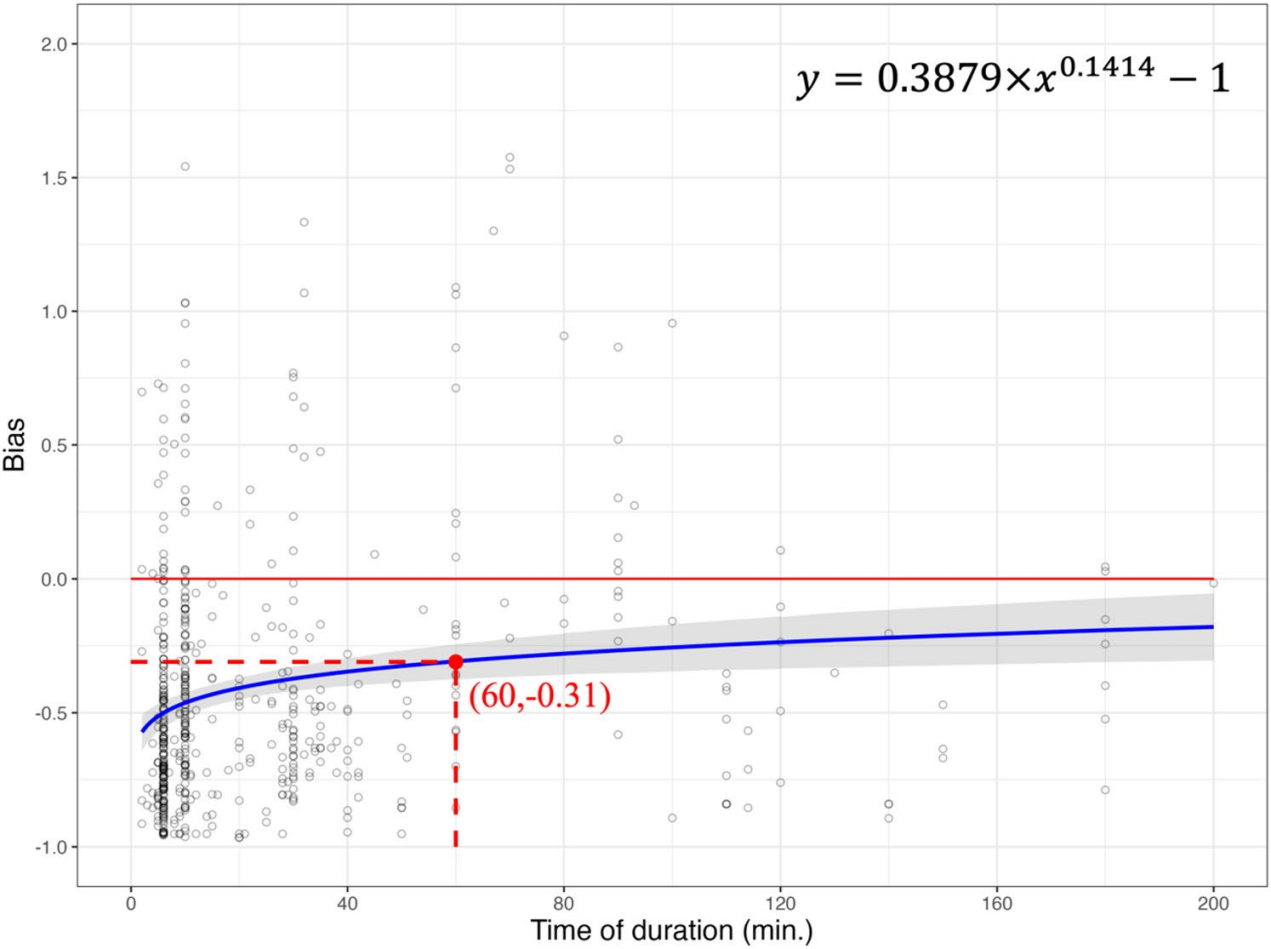
The relationship of duration on eBird checklists and bias. The power function (formula on the top right) estimated the non-linear relationship between duration and bias (blue solid line). Bias was derived from the results of estimated species richness in eBird and average observed species richness from BBS (black circle). Where bias = 0 (red solid horizontal line) indicates no differences among estimated species richness from eBird and average observed species richness from BBS, indicating eBird could record an average of 100% of the BBS species richness.

**Figure 4:**
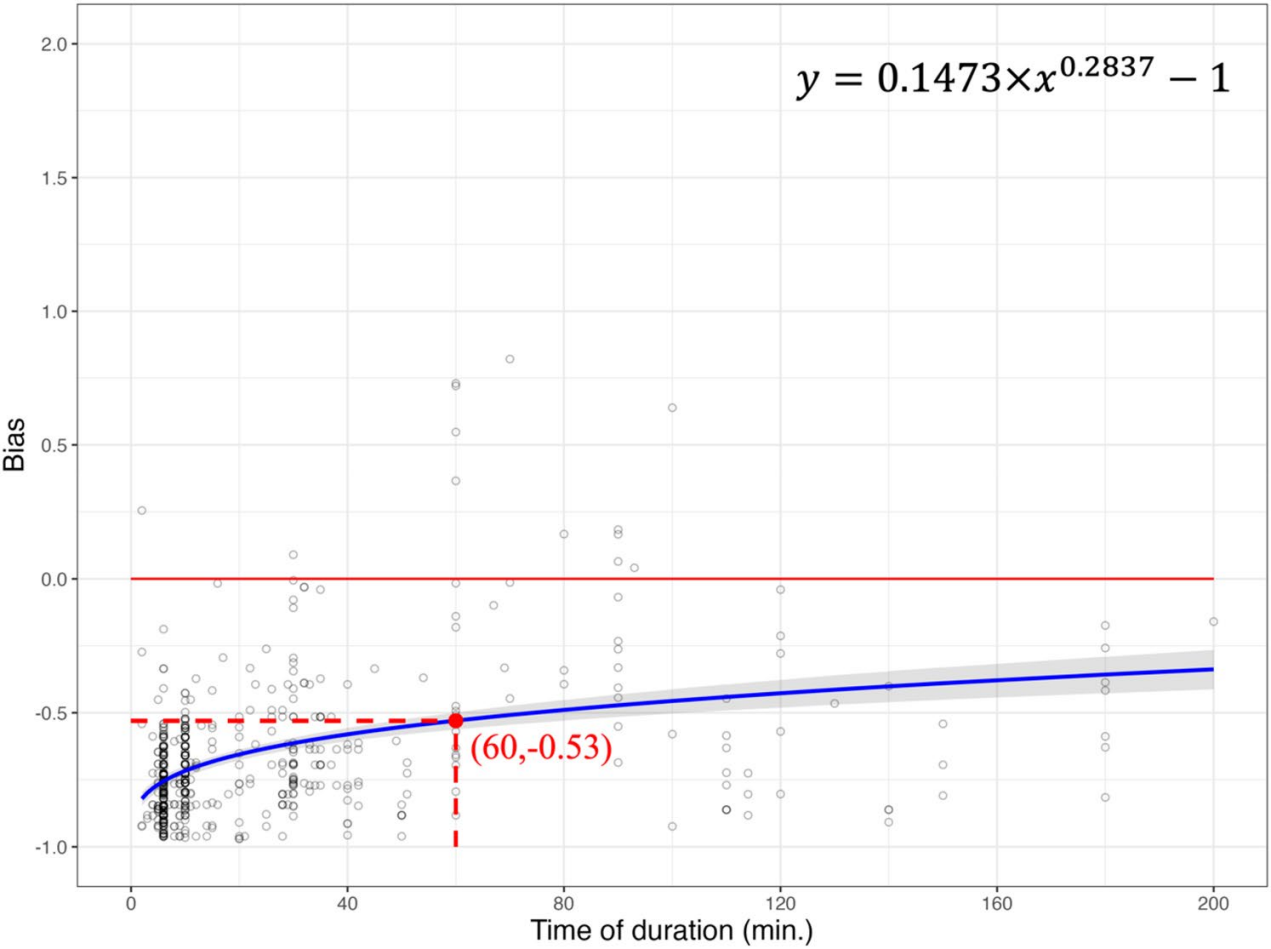
The relationship of duration on eBird checklists and bias. The power function (formula on the top right) estimated the non-linear relationship between duration and bias (blue solid line). Bias was derived from the results of observed species richness in eBird and average estimated species richness from BBS (black circle). Where bias = 0 (red solid horizontal line) indicates no differences among observed species richness from eBird and average estimated species richness from BBS, indicating eBird could record an average of 100% of the BBS species richness.

**Figure 5:**
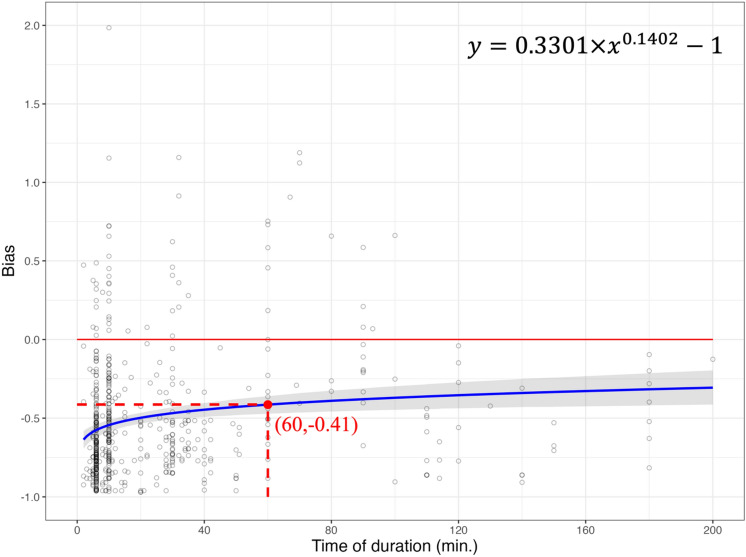
The relationship of duration on eBird checklists and bias. The power function (formula on the top right) estimated the non-linear relationship between duration and bias (blue solid line). Bias was derived from the results of estimated species richness in eBird and average estimated species richness from BBS (black circle). Where bias = 0 (red solid horizontal line) indicates no differences among estimated species richness from eBird and average estimated species richness from BBS, indicating eBird could record an average of 100% of the BBS species richness.

A non-linear power function by least squares approach explained the effect of duration on the bias (Tables 1, 2, 3 and 4). As the duration of eBird checklists increased from 1 to 200 min, both observed and estimated species richness approached the average species richness of BBS sites (Figs. 2, 3, 4 and 5). At 60-min of power function, bias was closer to zero (− 0.44 to − 0.31) after the Chao1 estimator was applied to observed species richness in the eBird dataset (Figs. 2 and 3). In other words, after using the Chao1 estimator, the ability of the eBird dataset to record the same species richness compared to the BBS dataset rose from 56 to 69%, indicating species richness from the eBird dataset was closer to average observed species richness from the BBS dataset after applying the Chao1 estimator. The standard error of parameters for the power function derived from the Chao1 estimator was relatively larger compared to without applying estimator (Tables 1 and 2), which reflected a wider confidence interval (Fig. 3).

**Table 1:** Parameter estimates from the power function by least squares method on the relationship of duration and bias (observed species richness of eBird vs. average observed species richness of BBS).

Parameter	Estimate	Standard error	*t* value	*p* value
a	0.1729	0.0151	11.43	** < 0.001**
b	0.2853	0.0250	11.45	** < 0.001**

The power function is depicted in Eq. (5) with parameters (a and b) to be estimated. *Residual standard error* 0.2692 on 562 degrees of freedom. Significant parameters are shown in bold.

**Table 2:** Parameter estimates from the power function by least squares method on the relationship of duration and bias (estimated species richness of eBird vs. average observed species richness of BBS).

Parameter	Estimate	Standard error	*t* value	*p* value
c	0.3879	0.0380	10.205	** < 0.001**
d	0.1414	0.0310	4.566	** < 0.001**

The power function is depicted in Eq. (6) with parameters (c and d) to be estimated. *Residual standard error* 0.4621 on 562 degrees of freedom. Significant parameters are shown in bold.

**Table 3:** Parameter estimates from the power function by least squares method on the relationship of duration and bias (observed species richness of eBird vs. average estimated species richness of BBS).

Parameter	Estimate	Standard error	*t* value	*p* value
e	0.1473	0.0129	11.39	** < 0.001**
f	0.2837	0.0250	11.33	** < 0.001**

The power function is depicted in Eq. (7) with parameters (e and f) to be estimated. *Residual standard error* 0.229 on 562 degrees of freedom. Significant parameters are shown in bold.

**Table 4:** Parameter estimates from the power function by least squares method on the relationship of duration and bias (estimated species richness of eBird vs. average estimated species richness of BBS).

Parameter	Estimate	Standard error	*t* value	*p* value
c	0.3301	0.0326	10.118	** < 0.001**
d	0.1402	0.0312	4.485	** < 0.001**

The power function is depicted in Eq. (8) with parameters (g and h) to be estimated. *Residual standard error* 0.3954 on 562 degrees of freedom. Significant parameters are shown in bold.

After implementing the Chao1 estimator in the BBS dataset, similarly, bias was closer to zero (− 0.53 to − 0.41) after the Chao1 estimator was applied to observed species richness in the eBird dataset (Figs. 4 and 5). In other words, the implementation of the Chao1 estimator increased the ability of the eBird dataset to record the same species richness against the BBS dataset rose from 47 to 59%. Again, the Chao1 estimators resulted in a relatively larger standard error of parameters when estimating the power function (Tables 3 and 4), which also reflected a wider confidence interval (Fig. 5).

When comparing observed species richness in the eBird dataset to average observed species richness in the BBS dataset at 60-min from the power function, the eBird dataset had a bias of − 0.44 (Fig. 2). That is, the eBird dataset collectively recorded an average of 56% of the BBS species richness at 60-min based on the Eq. (1) described in the methods section. When not interpreting the relationship between bias and duration through the power function (6–200 min.), 25 eBird checklists were able to record the same or more species richness as the BBS dataset (bias $\ge$ 0). However, according to the power function, the eBird dataset failed to record the same species richness as the BBS dataset (bias $= 0$) at the duration between 6 to 200 min.

When comparing the Chao1 estimator applied in the eBird dataset to average observed species richness in the BBS dataset at 60-min from the power function, the eBird dataset had a bias of − 0.31 (Fig. 3). That is, the eBird dataset collectively recorded an average of 69% of the BBS species richness at 60-min based on the Eq. (2) described in the methods section. Although the Chao1 estimator could calibrate the observed species richness from the eBird dataset, the eBird dataset was unable to reach the same number of species richness as the BBS dataset at 60-min (bias =  − 0.31) (Fig. 3). Despite the Chao1 estimator increased the ability of eBird to capture more species, the eBird dataset failed to record the same species richness as the BBS dataset (bias = 0) at the duration between 6 to 200 min according to the power function. A total of 76 eBird checklists were able to record the same or more species richness as the BBS dataset (bias $\ge$ 0) when disregarding the power function prediction.

After applying the Chao1 estimator in the BBS dataset, we found that the average estimated species richness in the BBS dataset produced a larger discrepancy for eBird at 60-min of power function (bias =  − 0.53 vs. − 0.44), compared to without applying the Chao1 estimator in the BBS dataset (Figs. 2 and 4). Even after applying the Chao1 estimator in the eBird dataset, eBird was only able to capture 59% of the same species richness compared to BBS (Fig. 5), which was less than without applying the Chao1 estimator in the BBS dataset (69%) (Fig. 3). Similar patterns were found without interpreting the bias vs. duration relationship through power function. We found that 13 eBird checklists with applying the Chao1 estimator in the BBS vs. 25 eBird checklists without applying the Chao1 estimator in the BBS, were able to record the same or more species richness as the BBS dataset (bias $\ge$ 0) (Figs. 2 and 4), while a total of 53 eBird checklists with the Chao1 applied in the BBS vs. 76 eBird checklists without the Chao1 applied in the BBS were able to record the same or more species richness as the BBS dataset (bias $\ge$ 0) (Figs. 3 and 5).

## Discussion

To address the effectiveness of species richness estimation, we made a comparison of bias after implementing the species richness estimator in the both eBird (semi-structured citizen science) and BBS (structured citizen science) datasets, and compared eBird with the BBS dataset at a 60-min cut-off point. As the result, the Chao1 estimator calibrated incomplete samples and increased the species richness in the eBird dataset against the original BBS dataset from 56 to 69%; and from 47 to 59% when compared to estimated BBS dataset. These results highlight the importance of taking into account the incomplete samples by using a species richness estimator, as the Chao1 estimator showed improvement in estimating species richness not only in eBird, but also in BBS. From museum-based bird data, Guralnick and Van Cleve ^44^ found an average 25.86% increase in species richness after implementing the Chao1 estimator. By comparing the effectiveness of the Chao1 estimator at an accumulated 2,500 min of the survey, the Chao1 estimator increased 22.47% of species richness according to the case study of the bird community in watershed area in India^45^. Chao estimators (Chao1 and Chao2) have been widely applied across various taxa to estimate the asymptote species richness. For example, the Chao1 estimator has increased 42.00% in average spider species richness across 16,920 min of accumulated samples^46^. Here, the following explanation in this discussion will be focusing on two main topics: (1) the possibility of biased results from the eBird dataset; (2) overestimation issues derived from the proportion of singleton species in the Chao1 estimation process.

When comparing species richness from different datasets, data quality control and validation are prerequisites^10,47–49^. In this study, we controlled various factors that may bias results developed from different survey methods, including (1) time of season; (2) time of day; (3) survey area within a 2 $\times$ 2 km area; (4) traveling distance; (5) removal of incomplete, unaccepted, and incidental eBird checklists; and (6) removal of group sharing checklists. Here, we aimed to focus on the comparison of species richness index between the two datasets. The completeness of an eBird checklist will influence the total reported species, thus resulting in the discrepancy of species richness measures. After controlling the potential factors that may bias results, the aim to address the effects of duration on the bias from two datasets will be more robust.

Analyzing the results of bias across a large continuous duration of survey effort will be more comprehensive and help researchers to understand the relationship between bias and duration^20^. Once the relationship between survey effort and relative species richness has been determined, it is crucial to compare different sources of data at a standardized sample size^48^. In this study, we presented a total duration of 200 min and compared it with the results of bias. Predicted by the power function, our results showed that at 60-min, the eBird dataset failed to record the same species richness as the BBS dataset. Underestimation of the mean may occur under the flaws of data collection process, resulting in biased estimates^12^. These apparent underestimates of species richness derived from the eBird dataset are likely due to the following reasons.

First, there is a higher likelihood to record more species across survey points in a BBS site compared to the eBird dataset. The BBS aims to monitor a large number of common bird species that regularly breed in a specific area^50^. To monitor bird species that may occupy a range of habitats, the BBS program was designed to cover all possible breeding bird species within a 2 $\times$ 2 km area by setting up 6 to 10 points. The unique geographic characteristics of Taiwan usually produce large changes in elevation over a short distance, thus creating closely spaced heterogeneous habitats. Therefore, a more heterogeneous habitat may result in a more diverse bird composition. In this study, despite the BBS monitoring program followed a point count survey protocol, a volunteer may record different bird species from each visit across 10 points within a site. Compared to the BBS dataset, the eBird dataset included stationary protocol (95.22%), which only retains bird records appearing in a fixed location. According to the best practices from eBird, it suggests that the distance traveling should not be longer than 30 m away from the starting point, which indicates a lower possibility to detect more species from different habitat types. To understand the potential influence of habitat type on biases, we further investigated the effect of BBS main habitat types on biases and compared with different species richness estimator implementation strategy in eBird and BBS datasets. The results showed no strong effect on bias among habitat types (i.e., forest, agriculture, and all-combined). One possible explanation is that with the mosaic nature of habitat in low-elevation sites in Taiwan, a site defined as forest habitat may still contain different degrees of agricultural habitat, which prevents us to elucidate the relation in this scale (Supplementary Figure S3 online).

Second, level of bird activities varies with weather conditions^51^. From a study in North America, Robbins^51^ demonstrated that half of the families of birds had reduced population estimates during light rain. To follow the best practice of collecting data, the BBS monitoring program restricts the surveys only conducted in good weather conditions. On the contrary, eBird observers might conducted surveys in bad weather conditions (e.g., heavy rain), resulting in less probability to record bird species.

Third, skills of identification influence the detection probability of birds. A well-experienced observer tends to record more species than a relatively less experienced observer^52^. Uncommon species may be overlooked simply because they are challenging to be detected or recognized (i.e., imperfect species detectability). For example, some uncommon species lack key features or distinctive song pattern, resulting in lower detection probability^53,54^. Thus, less experienced observers in eBird may not detect rare or unrecognizable species. A species identification training program can help citizen scientists to detect more species^55^. For instance, volunteers that participate in New York Breeding Bird Atlas showed an increase in identification skills after attending training programs^6^. Moreover, the false-positives rate declined significantly as the observer’s identification skill level increased^52^. In Taiwan, the BBS program held training programs at least twice a year for volunteers to acquire survey skills, such as practical instructions on conducting surveys, and identification tips for breeding birds. We speculate volunteers from BBS will increase their identification skills after attending the training programs. By contrast, untrained observers with varying identification skills in eBird may result in biased estimate of species richness.

We also found that after applying the Chao1 estimator in eBird checklists with lower duration checklists (e.g., < 20 min), the number of checklists with estimated species richness that outperform BBS also increased (see Fig. 2 vs. 3, and Fig. 4 vs. 5). The flaws stem from the estimation process would likely produce estimation over the mean^12^. The Chao1 estimator specifies the number of singletons from a reference sample with rare or undetected species^17^. When a large number of singleton species remains in a reference sample, the output estimation result will likely be biased. Among species richness estimators, the Chao1 estimator is particularly sensitive to the number of singletons from a reference sample. It has been found that more singletons would appear in a low sampling effort than in a larger sampling effort^56^. Therefore, we investigated the relationship between duration and percentage of singleton species in eBird checklists. As a result, as duration decreased, the percentage of singletons increased significantly in response (see Supplementary Table S3 and Fig. S1 online). Further, we found the value of bias had a positive relationship with the percentage of singleton species. In other words, as the percentage of singleton species increases, the value of bias will increase in response (see Supplementary Table S4 and Fig. S2 online). Our results showed that the possible bias derived from the estimation process may result from the number of singleton species. Therefore, when singleton species are abundant from a reference sample, the Chao1 estimator tends to outperform the dataset that we are comparing.

It has been found that in semi-structured citizen science, the number of singleton species presents an issue more than in structured citizen science. Soroye, et al.^6^ explored the accuracy of species richness in a semi-structured citizen science dataset–eButterfly. When using eButterfly to estimate the asymptote species richness, the outcome from estimation was more accurate when rare species were excluded^6^. Although non-parametric species estimation methods make no assumption on species abundance distribution, variable forms of distribution from samples can still affect the effectiveness of these estimators^57,58^. In addition, the survey effort over which samples are collected may affect the shape of species abundance distributions^59^. In general, samples collected with greater sampling efforts usually produce more accurate estimates than lesser sampling efforts^60^. To produce a more reliable estimate of species richness, further studies should take into account the effect of the number of singleton species, particularly in a low-effort reference sample from semi-structured citizen science. It should be noted that we only estimated species richness based on each individual eBird checklist in this study. Thus, singleton species might occupy a large proportion of the species list, resulting in a biased estimate.

We suggest a more robust way to increase the ability to use semi-structured citizen science when structured citizen science is absent. One can take into account samples with a larger sampling effort (e.g., duration, number of individuals). Nevertheless, insufficient samples may occur in some remote or distant areas^61,62^, since checklists collected in semi-structured citizen science exhibit a spatial bias towards more densely populated areas or interesting sites (e.g., hotspots)^63–65^. Else, another way to increase power and reduce uncertainty around associated outcomes is to combine checklists and datasets as a whole. When combining individual observation with additional observations, reference samples may show the low occurrence of singleton species and thus improve the species richness estimation outcome.

Here, we would like to point out potential future research directions. Datasets integration may be a practical strategy because it may help to offset missing visits in structured citizen science, maximizing the potential use of semi-structured citizen science as an auxiliary dataset. Data integration has been documented in the context of species distribution modeling settings^66^. Although our results showed that eBird failed to record the same species richness as BBS at 60 min cut-off point in power function. Careful use of eBird accompanying BBS may assist the Chao1 estimator in better predicting species richness. In this study, despite we included BBS sites with over eight years of surveys, some BBS sites did not conduct consecutive yearly visits (Supplementary Table S2), which may impact the estimates of species richness.

In summary, semi-structured citizen science has aroused a prominent mechanism for collecting biodiversity data in recent decades. We investigated the non-linear relationship of bias from a large extent of duration for each individual eBird checklist. The Chao1 estimator increased the number of recorded species from 56 to 69% in the eBird dataset compared to average observed species richness in the BBS dataset, and from 47 to 59% when compared to the estimated BBS dataset. However, our results showed a discrepancy in species richness between the eBird and BBS datasets. According to power function, the eBird dataset failed to record the same number of species as the BBS dataset. This biased outcome might result from habitat heterogeneity in a BBS site, different levels of identification skills, incomplete samples, and weather conditions. We also found that the presence of singleton species may bias the accuracy of species richness estimates, especially often found in lower duration samples.

Finally, we conclude that further studies from semi-structured citizen science should: (1) always account for the impact of incomplete samples derived from uneven duration, as well as the effect of imperfect detection probability from different observers; (2) always compare biodiversity measures at a standardized sampling effort after controlling potential biases from different survey protocols; and (3) when one attempts to apply the Chao1 estimator, more attention should be paid to the percentage of singleton species, which may bias the estimation outcome (e.g., overestimation). According to our findings, we provide the following priorities for the future use of structured and semi-structured citizen science in monitoring species richness. First, it would be ideal to incorporate a species richness estimator on sufficient samples from structured citizen science. Second, if structured citizen science is absent, one may apply species richness estimator on semi-structured citizen science data to accommodate incomplete samples. We provided step-by-step instruction along with annotations in R platform regrading applying the Chao1 estimator in eBird (see Supplementary online). Once the estimates of species richness are calibrated, and the effect of singletons are dealt with, better conservation actions can be established for areas where biodiversity has been impacted.

## Supplementary Information

Supplementary Information.

Supplementary Information.

## Data Availability

The datasets generated during the current study are available in Dataverse. eBird (https://science.ebird.org/en/use-ebird-data/download-ebird-data-products); BBS (https://sites.google.com/a/birds-tesri.twbbs.org/bbs-taiwan/bbs-zi-liao-shen-qing).
