# Supplementary material for: Comparing avian species richness estimates from structured and semi-structured citizen science data
Source: Sci Rep. 2023 Jan 21;13:1214. doi: 10.1038/s41598-023-28064-7 (PMC9867693; doi:10.1038/s41598-023-28064-7)
Supplement: Supplementary file 2 — Supplementary Information. [file 41598_2023_28064_MOESM2_ESM.docx]

# Supplementary Information

**Table S1** The original bird species reported from the Breeding Bird Survey Taiwan (BBS) and eBird datasets from 2010 to 2017.

*Note: The species list was from the original dataset that has not applied the filtering steps in the method section. Where “1” represents the species reported from the datasets, “NA” represents the species that were not reported from the datasets.

| **Common Name** | **Scientific Name** | **BBS** | **eBird** |
| --- | --- | --- | --- |
| Barred Buttonquail | *Turnix suscitator* | 1 | 1 |
| Long-tailed Shrike | *Lanius schach* | 1 | 1 |
| White-bellied Erpornis | *Erpornis zantholeuca* | 1 | 1 |
| Large Cuckooshrike | *Coracina macei* | 1 | 1 |
| Gray-chinned Minivet | *Pericrocotus solaris* | 1 | 1 |
| Taiwan Yellow Tit | *Machlolophus holsti* | 1 | 1 |
| Green-backed Tit | *Parus monticolus* | 1 | 1 |
| Coal Tit | *Periparus ater* | 1 | 1 |
| Chestnut-bellied Tit | *Sittiparus castaneoventris* | 1 | 1 |
| Alpine Accentor | *Prunella collaris* | 1 | 1 |
| Striated Swallow | *Cecropis striolata* | 1 | 1 |
| Asian House-Martin | *Delichon dasypus* | 1 | 1 |
| Barn Swallow | *Hirundo rustica* | 1 | 1 |
| Pacific Swallow | *Hirundo tahitica* | 1 | 1 |
| Gray-throated Martin | *Riparia chinensis* | 1 | 1 |
| Oriental Pratincole | *Glareola maldivarum* | 1 | 1 |
| **Table S1** (continued) |  |  |  |
| **Common Name** | **Scientific Name** | **BBS** | **eBird** |
| Bronzed Drongo | *Dicrurus aeneus* | 1 | 1 |
| Black Drongo | *Dicrurus macrocercus* | 1 | 1 |
| Black-naped Monarch | *Hypothymis azurea* | 1 | 1 |
| Japanese Paradise-Flycatcher | *Terpsiphone atrocaudata* | 1 | 1 |
| Rufous-capped Babbler | *Cyanoderma ruficeps* | 1 | 1 |
| Black-necklaced Scimitar-Babbler | *Megapomatorhinus erythrocnemis* | 1 | 1 |
| Taiwan Scimitar-Babbler | *Pomatorhinus musicus* | 1 | 1 |
| Fire-breasted Flowerpecker | *Dicaeum ignipectus* | 1 | 1 |
| Plain Flowerpecker | *Dicaeum minullum* | 1 | 1 |
| White-backed Woodpecker | *Dendrocopos leucotos* | 1 | 1 |
| Gray-headed Woodpecker | *Picus canus* | 1 | 1 |
| Gray-capped Woodpecker | *Yungipicus canicapillus* | 1 | 1 |
| Common Kingfisher | *Alcedo atthis* | 1 | 1 |
| Crested Myna | *Acridotheres cristatellus* | 1 | 1 |
| Oriental Skylark | *Alauda gulgula* | 1 | 1 |
| Taiwan Barwing | *Actinodura morrisoniana* | 1 | 1 |
| Morrison's Fulvetta | *Alcippe morrisonia* | 1 | 1 |
| Taiwan Hwamei | *Garrulax taewanus* | NA | 1 |
| White-eared Sibia | *Heterophasia auricularis* | 1 | 1 |

**Table S1** (continued)

| **Common Name** | **Scientific Name** | **BBS** | **eBird** |
| --- | --- | --- | --- |
| Rusty Laughingthrush | *Ianthocincla poecilorhyncha* | 1 | 1 |
| Rufous-crowned Laughingthrush | *Ianthocincla ruficeps* | 1 | 1 |
| Steere's Liocichla | *Liocichla steerii* | 1 | 1 |
| White-whiskered Laughingthrush | *Trochalopteron morrisonianum* | 1 | 1 |
| White-breasted Waterhen | *Amaurornis phoenicurus* | 1 | 1 |
| Eurasian Moorhen | *Gallinula chloropus* | 1 | 1 |
| Slaty-legged Crake | *Rallina eurizonoides* | 1 | 1 |
| Ruddy-breasted Crake | *Zapornia fusca* | 1 | 1 |
| Taiwan Yuhina | *Yuhina brunneiceps* | 1 | 1 |
| Swinhoe's White-eye | *Zosterops simplex* | 1 | 1 |
| Lowland White-eye | *Zosterops meyeni* | 1 | 1 |
| Greater Painted-Snipe | *Rostratula benghalensis* | 1 | 1 |
| Taiwan Barbet | *Psilopogon nuchalis* | 1 | 1 |
| Rufous-faced Warbler | *Abroscopus albogularis* | 1 | 1 |
| Yellowish-bellied Bush Warbler | *Horornis acanthizoides* | 1 | 1 |
| Brownish-flanked Bush Warbler | *Horornis fortipes* | 1 | 1 |
| White Wagtail | *Motacilla alba* | 1 | 1 |
| Little Forktail | *Enicurus scouleri* | 1 | 1 |
| Snowy-browed Flycatcher | *Ficedula hyperythra* | 1 | 1 |
| Ferruginous Flycatcher | *Muscicapa ferruginea* | 1 | 1 |
| **Table S1** (continued) |  |  |  |
| **Common Name** | **Scientific Name** | **BBS** | **eBird** |
| Taiwan Whistling-Thrush | *Myophonus insularis* | 1 | 1 |
| Vivid Niltava | *Niltava vivida* | 1 | 1 |
| Plumbeous Redstart | *Phoenicurus fuliginosus* | 1 | 1 |
| White-browed Bush-Robin | *Tarsiger indicus* | 1 | 1 |
| Taiwan Shortwing | *Brachypteryx goodfellowi* | 1 | 1 |
| Collared Bush-Robin | *Tarsiger johnstoniae* | 1 | 1 |
| Scaly Thrush | *Zoothera dauma* | 1 | 1 |
| Taiwan Fulvetta | *Fulvetta formosana* | 1 | 1 |
| Vinous-throated Parrotbill | *Sinosuthora webbiana* | 1 | 1 |
| Golden Parrotbill | *Suthora verreauxi* | 1 | 1 |
| Little Grebe | *Tachybaptus ruficollis* | 1 | 1 |
| Taiwan Cupwing | *Pnoepyga formosana* | 1 | 1 |
| Eurasian Wren | *Troglodytes troglodytes* | 1 | 1 |
| Cattle Egret | *Bubulcus ibis* | 1 | 1 |
| Striated Heron | *Butorides striata* | 1 | 1 |
| Little Egret | *Egretta garzetta* | 1 | 1 |
| Pacific Reef-Heron | *Egretta sacra* | 1 | 1 |
| Malayan Night-Heron | *Gorsachius melanolophus* | 1 | 1 |
| Cinnamon Bittern | *Ixobrychus cinnamomeus* | 1 | 1 |
| Yellow Bittern | *Ixobrychus sinensis* | 1 | 1 |
| **Table S1** (continued) |  |  |  |
| **Common Name** | **Scientific Name** | **BBS** | **eBird** |
| Black-crowned Night-Heron | *Nycticorax nycticorax* | 1 | 1 |
| Brown Noddy | *Anous stolidus* | NA | 1 |
| Bridled Tern | *Onychoprion anaethetus* | NA | 1 |
| Sooty Tern | *Onychoprion fuscatus* | NA | 1 |
| Roseate Tern | *Sterna dougallii* | NA | 1 |
| Black-naped Tern | *Sterna sumatrana* | NA | 1 |
| Little Tern | *Sternula albifrons* | 1 | 1 |
| Great Crested Tern | *Thalasseus bergii* | NA | 1 |
| Chinese Crested Tern | *Thalasseus bernsteini* | NA | 1 |
| Crested Goshawk | *Accipiter trivirgatus* | 1 | 1 |
| Besra | *Accipiter virgatus* | 1 | 1 |
| Black-winged Kite | *Elanus caeruleus* | 1 | 1 |
| Black Eagle | *Ictinaetus malaiensis* | 1 | 1 |
| Black Kite | *Milvus migrans* | 1 | 1 |
| Mountain Hawk-Eagle | *Nisaetus nipalensis* | 1 | 1 |
| Crested Serpent-Eagle | *Spilornis cheela* | 1 | 1 |
| Pheasant-tailed Jacana | *Hydrophasianus chirurgus* | 1 | 1 |
| Russet Sparrow | *Passer cinnamomeus* | 1 | 1 |
| Eurasian Tree Sparrow | *Passer montanus* | 1 | 1 |
| Brown Dipper | *Cinclus pallasii* | 1 | 1 |
| **Table S1** (continued) |  |  |  |
| **Common Name** | **Scientific Name** | **BBS** | **eBird** |
| Asian Emerald Dove | *Chalcophaps indica* | 1 | 1 |
| Ashy Wood-Pigeon | *Columba pulchricollis* | 1 | 1 |
| Philippine Cuckoo-Dove | *Macropygia tenuirostris* | 1 | 1 |
| Black-chinned Fruit-Dove | *Ptilinopus leclancheri* | NA | 1 |
| Spotted Dove | *Streptopelia chinensis* | 1 | 1 |
| Oriental Turtle-Dove | *Streptopelia orientalis* | 1 | 1 |
| Red Collared-Dove | *Streptopelia tranquebarica* | 1 | 1 |
| Whistling Green-Pigeon | *Treron formosae* | 1 | 1 |
| White-bellied Green-Pigeon | *Treron sieboldii* | 1 | 1 |
| Eurasian Nuthatch | *Sitta europaea* | 1 | 1 |
| Chestnut Munia | *Lonchura atricapilla* | 1 | 1 |
| Scaly-breasted Munia | *Lonchura punctulata* | 1 | 1 |
| White-rumped Munia | *Lonchura striata* | 1 | 1 |
| Large-billed Crow | *Corvus macrorhynchos* | 1 | 1 |
| Gray Treepie | *Dendrocitta formosae* | 1 | 1 |
| Eurasian Jay | *Garrulus glandarius* | 1 | 1 |
| Eurasian Nutcracker | *Nucifraga caryocatactes* | 1 | 1 |
| Taiwan Blue-Magpie | *Urocissa caerulea* | 1 | 1 |
| Flamecrest | *Regulus goodfellowi* | 1 | 1 |
| Golden-headed Cisticola | *Cisticola exilis* | 1 | 1 |
| **Table S1** (continued) |  |  |  |
| **Common Name** | **Scientific Name** | **BBS** | **eBird** |
| Zitting Cisticola | *Cisticola juncidis* | 1 | 1 |
| Striated Prinia | *Prinia crinigera* | 1 | 1 |
| Yellow-bellied Prinia | *Prinia flaviventris* | 1 | 1 |
| Plain Prinia | *Prinia inornata* | 1 | 1 |
| Black-naped Oriole | *Oriolus chinensis* | 1 | 1 |
| Maroon Oriole | *Oriolus traillii* | 1 | 1 |
| Black-throated Tit | *Aegithalos concinnus* | 1 | 1 |
| House Swift | *Apus nipalensis* | 1 | 1 |
| Silver-backed Needletail | *Hirundapus cochinchinensis* | 1 | 1 |
| Taiwan Rosefinch | *Carpodacus formosanus* | 1 | 1 |
| Gray-headed Bullfinch | *Pyrrhula erythaca* | 1 | 1 |
| Brown Bullfinch | *Pyrrhula nipalensis* | 1 | 1 |
| Taiwan Partridge | *Arborophila crudigularis* | 1 | 1 |
| Taiwan Bamboo-Partridge | *Bambusicola sonorivox* | 1 | 1 |
| Swinhoe's Pheasant | *Lophura swinhoii* | 1 | 1 |
| Ring-necked Pheasant | *Phasianus colchicus* | 1 | 1 |
| Mikado Pheasant | *Syrmaticus mikado* | 1 | 1 |
| Dusky Fulvetta | *Schoeniparus brunneus* | 1 | 1 |
| Mandarin Duck | *Aix galericulata* | 1 | 1 |
| Eastern Spot-billed Duck | *Anas zonorhyncha* | 1 | 1 |
| **Table S1** (continued) |  |  |  |
| **Common Name** | **Scientific Name** | **BBS** | **eBird** |
| Taiwan Bush-Warbler | *Locustella alishanensis* | 1 | 1 |
| Lesser Coucal | *Centropus bengalensis* | 1 | 1 |
| Oriental Cuckoo | *Cuculus optatus* | 1 | 1 |
| Large Hawk-Cuckoo | *Hierococcyx sparverioides* | 1 | 1 |
| Brown-eared Bulbul | *Hypsipetes amaurotis* | 1 | 1 |
| Black Bulbul | *Hypsipetes leucocephalus* | 1 | 1 |
| Light-vented Bulbul | *Pycnonotus sinensis* | 1 | 1 |
| Styan's Bulbul | *Pycnonotus taivanus* | 1 | 1 |
| Collared Finchbill | *Spizixos semitorques* | 1 | 1 |

**Table S2** Summary of the selected 92 BBS sites from this study, including main habitat type, number of visits, and total survey duration recorded from 2010 to 2017. “A” denotes sites located in low-elevation (<1,000 meters a.s.l.); “B” denotes sites located in mid-elevation (1,000–2,500 meters a.s.l.); “C” denotes sites located in high-elevation (>2,500 meters a.s.l). Volunteers recorded habitat types at each BBS point. The main habitat type of each BBS site was determined as the majority of habitat types in all BBS points of the BBS site.

| Site ID | Main habitat type | Number of visits from 2010 to 2017 | Total survey duration (min.) |
| --- | --- | --- | --- |
| A01-02 | Forest | 14 | 1110 |
| A02-06 | Agriculture | 2 | 120 |
| A03-07 | Agriculture | 6 | 360 |
| A03-21 | Forest | 3 | 180 |
| A04-09 | Developed | 9 | 540 |
| A04-16 | Wetland | 11 | 660 |
| A04-18 | Developed | 13 | 780 |
| A04-19 | Forest | 11 | 660 |
| A04-20 | Forest | 14 | 840 |
| A04-21 | Forest | 11 | 660 |
| A04-22 | Forest | 6 | 360 |
| A04-23 | Forest | 13 | 780 |
| A04-24 | Forest | 12 | 720 |
| A04-26 | Forest | 8 | 480 |
| A04-27 | Developed | 3 | 180 |
| A04-28 | Developed | 14 | 840 |
| A04-31 | Agriculture | 10 | 600 |
| A04-34 | Developed | 9 | 540 |
| A04-41 | Forest | 7 | 420 |
| A04-43 | Developed | 12 | 720 |
| A04-49 | Forest | 10 | 600 |
| A07-10 | Forest | 6 | 360 |
| A09-13 | Forest | 12 | 720 |
| A09-15 | Developed | 12 | 720 |
| A09-29 | Agriculture | 11 | 660 |
| A09-31 | Agriculture | 9 | 540 |
| A09-45 | Developed | 12 | 720 |
| A09-46 | Forest | 12 | 720 |
| A09-52 | Forest | 9 | 540 |
| A16-04 | Forest | 12 | 1080 |
| A17-04 | Developed | 15 | 1080 |
| A18-04 | Forest | 12 | 720 |
| A18-07 | Grassland | 4 | 240 |
| A19-02 | Forest | 15 | 1080 |
| A20-02 | Forest | 9 | 810 |
| A20-04 | Forest | 15 | 1170 |
| A27-33 | Agriculture | 8 | 480 |
| A29-03 | Forest | 16 | 1230 |
| A29-13 | Agriculture | 2 | 120 |
| A29-17 | Forest | 14 | 840 |
| A29-20 | Wetland | 13 | 780 |
| A29-21 | Grassland | 12 | 720 |
| A32-02 | Forest | 16 | 960 |
| A32-03 | Agriculture | 16 | 960 |
| A33-01 | Agriculture | 16 | 1230 |
| A33-02 | Agriculture | 16 | 1230 |
| A33-04 | Agriculture | 8 | 750 |
| A33-07 | Wetland | 16 | 1230 |
| A33-08 | Forest | 17 | 1200 |
| A33-14 | Agriculture | 14 | 840 |
| A33-15 | Agriculture | 14 | 840 |
| A33-18 | Agriculture | 12 | 720 |
| A33-23 | Forest | 2 | 120 |
| A33-26 | Agriculture | 14 | 840 |
| A33-27 | Forest | 10 | 600 |
| A34-08 | Forest | 15 | 1170 |
| A34-22 | Developed | 11 | 660 |
| A34-38 | Forest | 12 | 720 |
| A34-40 | Developed | 14 | 840 |
| A34-49 | Forest | 9 | 540 |
| A35-02 | Agriculture | 4 | 240 |
| A35-03 | Agriculture | 17 | 1020 |
| A35-09 | Forest | 9 | 540 |
| A35-10 | Agriculture | 5 | 300 |
| A35-16 | Forest | 8 | 480 |
| A35-17 | Agriculture | 2 | 120 |
| A36-15 | Forest | 14 | 840 |
| A40-17 | Forest | 6 | 360 |
| B06-01 | Forest | 12 | 990 |
| B10-01 | Forest | 16 | 1230 |
| B10-03 | Forest | 4 | 240 |
| B11-01 | Forest | 16 | 1140 |
| B14-01 | Forest | 16 | 1140 |
| B16-01 | Forest | 16 | 1230 |
| B21-01 | Forest | 10 | 870 |
| B28-06 | Forest | 2 | 120 |
| B29-02 | Forest | 10 | 600 |
| B30-01 | Forest | 14 | 1110 |
| B30-02 | Forest | 16 | 1230 |
| B30-04 | Forest | 16 | 1230 |
| B30-07 | Forest | 10 | 600 |
| B32-10 | Forest | 15 | 900 |
| B32-11 | Forest | 13 | 780 |
| B33-02 | Forest | 6 | 360 |
| B35-01 | Forest | 10 | 600 |
| B38-07 | Forest | 8 | 480 |
| C14-03 | Forest | 11 | 1020 |
| C16-01 | Forest | 12 | 720 |
| C30-02 | Grassland | 16 | 960 |
| C30-03 | Forest | 12 | 720 |
| C30-04 | Forest | 12 | 720 |
| C37-04 | Forest | 12 | 990 |

**Table S3** Estimates for the coefficient on linear regression analysis on the relationship of duration (min.) and percentage of singleton species (%) from eBird checklists. A total of 564 checklists were included in this analysis. Residual standard error was 28.12 on 562 degrees of freedom; adjusted R-squared was 0.1076 and F-statistic was 68.87 on 1 and 562 DF.

|  | Estimate | Standard error | t–value | p–value |
| --- | --- | --- | --- | --- |
| Intercept | 51.3687 | 1.4759 | 34.8050 | < 0.001*** |
| Duration | -0.2854 | 0.0344 | -8.2990 | < 0.001*** |

**Table S4** Estimates for the coefficient on linear regression analysis on the relationship of percentage of singleton species (%) and bias. A total of 564 checklists were included in this analysis. Residual standard error was 0.2714 on 562 degrees of freedom; adjusted R-squared was 0.1684 and F-statistic was 115 on 1 and 562 DF.

|  | Estimate | Standard error | t–value | p–value |
| --- | --- | --- | --- | --- |
| Intercept | 0.0041 | 0.0204 | 0.2010 | 0.84 |
| Percentage of singelton | 0.0041 | 0.0003 | 10.7240 | < 0.001*** |


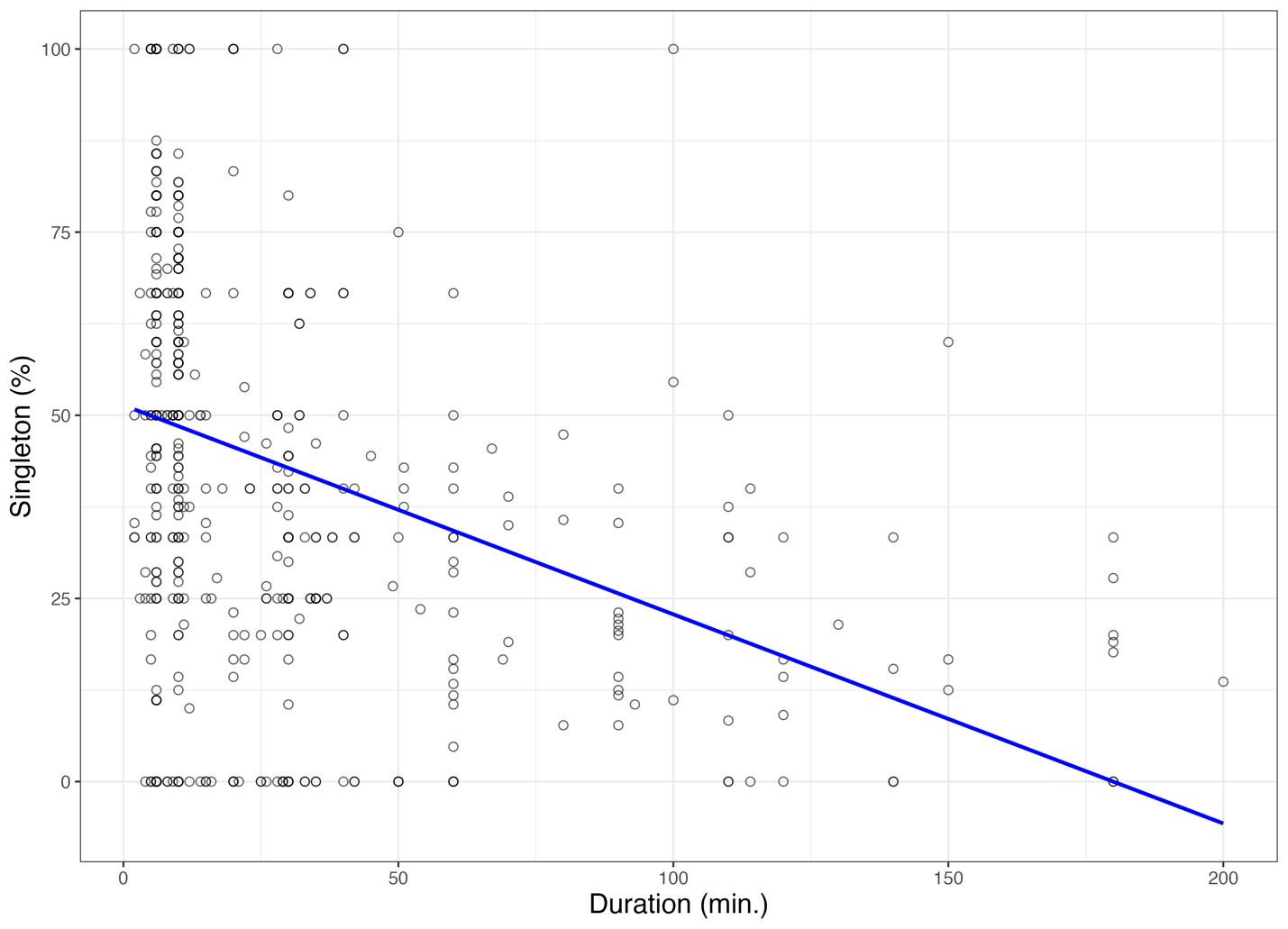


**Figure S1** Relationship between the percentage of singleton species and duration from 564 eBird checklists.


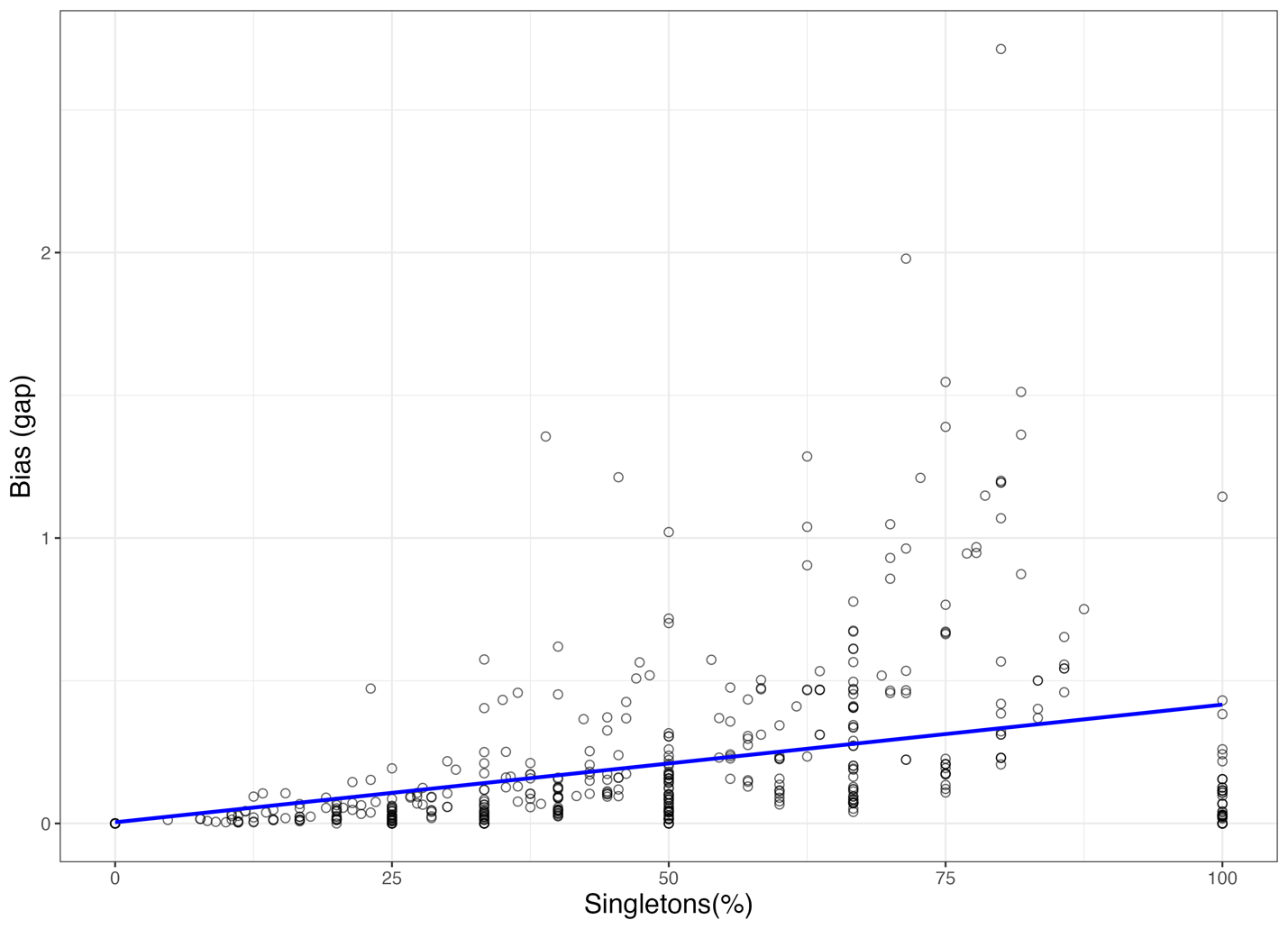


**Figure S2** Relationship between the percentage of singleton species and bias. Bias (gap) indicated here was calculated in the following method: the outcome of bias after species richness estimation in the eBird dataset subtracts the outcome of bias before species richness estimation in the eBird dataset. Species richness from eBird was applied as checklist-based, while species richness from BBS was compiled from years of visits recorded from 2010 to 2017.


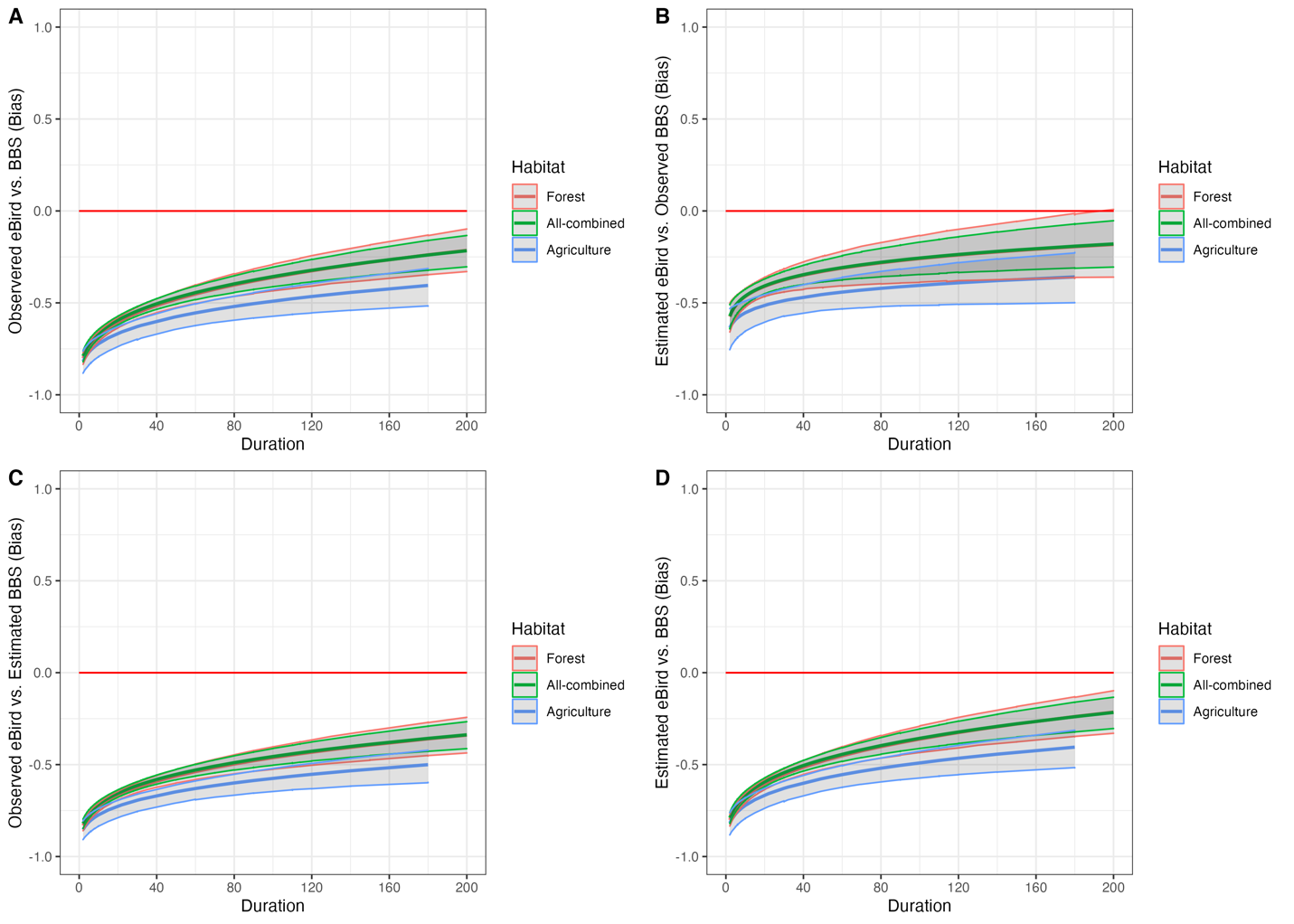


**Figure S3** The relationship between duration and estimated bias among three BBS habitat types (i.e., forest, all-combined, and agriculture). Where bias = 0 (red solid horizontal line) indicates no differences among species richness from eBird and BBS, indicating eBird could record an average of 100% of the BBS species richness. Bias value (y-axis) in panel A indicates the species richness differences compared by observed eBird vs. observed BBS; in panel B, bias was calculated by estimated eBird vs. observed BBS; in panel C, bias was calculated by observed BBS vs. estimated BBS; in panel D, bias was calculated by estimated eBird vs. estimated BBS. The detailed calculation is described in the methods section.
